# Decoction of heat-clearing, detoxifying and blood stasis removing relieves acute soft tissue injury via modulating miR-26b-5p/COX2 axis to inhibit inflammation

**DOI:** 10.1042/BSR20201981

**Published:** 2020-12-21

**Authors:** Shunwan Jiang, Zhi Chen, Wenqiang Lai, Qingchun Mai, Dayu Chen, Shufen Sun, Yong Zhang

**Affiliations:** Department of Orthopedics, The Fourth Clinical Medical College of Guangzhou University of Chinese Medicine, Shenzhen 518033, China

**Keywords:** COX2, heat-clearing, detoxifying and blood stasis removing decoction, inflammation, miR-26b-5p, Traditional Chinese medicine

## Abstract

Traditional Chinese medicine (TCM), such as Huanglian-Jie-Du-Tang, a heat-clearing and detoxifying decoction is beneficial for alleviation of inflammation-related diseases. The objective of the present study is to uncover the effect and mechanism of heat-clearing, detoxifying and blood stasis removing decoction (HDBD) on the treatment of acute soft tissue injury (STI) which is characterized with excessive inflammatory cascade at the onset. Male Sprague–Dawley (SD) rats with hammer beating served as the *in vivo* models of acute STI. Hematoxylin–Eosin (HE) staining was used for histopathology assessment. The levels of inflammatory factors, including prostaglandin E2 (PGE2), tumor necrosis factor-α (TNF-α), interleukin (IL)-1t and IL-6 were measured by enzyme-linked immunosorbent assay (ELISA). Human dermal microvascular endothelium cell line HMEC-1 and rat vascular endothelium cell line RAOEC were used to explore the mechanism *in vitro*. Luciferase gene reporter assay was applied to determine the relationship between miR-26b-5p and Cyclo-oxygenase 2 (COX2). The results showed that HDBD intervention significantly reduced the temperature difference between the healthy side and affected side of rats with hammer beating, together with the decreased levels of COX2, PGE2, TNF-α, IL-6 and IL-1β, and the increased level of miR-26b-5p. In mechanism, miR-26b-5p targeted COX2 and decreased its expression, leading to significant decreases in the levels of PGE2, TNF-α and IL-6 in RAOEC and HMEC-1 cells. In addition, miR-26b-5p inhibition impaired the effects of HDBD on the suppression of PGE2, TNF-α, IL-6 and IL-1β *in vitro*. In conclusion, the present study revealed that HDBD relieved acute STI via modulating miR-26b-5p/COX2 axis to inhibit inflammation.

## Introduction

Acute soft tissue injury (STI) is a common exercise injury in clinic, imposing a large burden on society due to the direct healthcare costs and time lost from productive employment [1,2]. Acute STI is characterized by aseptic inflammation and causes injuries in several manners, such as local edema, bruising, pain, muscle fiber fracture and petechiae [3,4]. Inflammation and pain reach at peak following the first 2 days of the injury and then decline quickly [5,6]. Noticeably, targeting the excessive inflammatory cascade has been considered as an effective method to improve acute STI [7,8].

Cyclo-oxygenase 2 (COX2), also called as prostaglandin-endoperoxide synthase 2 (PTGS2), serves as a crucial inflammation factor as same as tumor necrosis factor-α (TNF-α), interleukin (IL)-6 (IL-6) and IL-1β [9,10]. Increased evidence has shown that COX2 inhibitors (coxibs) display a good curative effect on the improvement of acute STI [2,11,12]. Prostaglandin E2 (PGE2), an arachidonic acid metabolite, serves as an inflammatory mediator in STI [8,13,14]. Through binding to one of the four downstream receptors (EP1–EP4), which belong to the superfamily of G protein-coupled receptors, PGE2 is closely involved in the inflammatory responses [15,16]. Targeting COX2 and PGE2 might be meaningful in inhibition of the excessive inflammation and the following alleviation of acute STI.

Traditional Chinese medicine (TCM) refers to the herbs and formulae which are made based on the Five Elements theory, including Metal, Wood, Water, Fire and Earth. Up to now, TCM has been applied for few thousand years in China and other Southeast Asian countries to prevent or cure almost all types of diseases, including the inflammation-related diseases [17–19]. Huanglian-Jie-Du-Tang, a decoction made up of heat-clearing and detoxifying TCM-relevant herbs such as *Coptis chinensis, Cortex Phellodendri, Scutellaria baicalensis* and *Fructus gardenia* has been identified to inhibit inflammation in inflamed rats, accompanied with decreased levels of macrophage inflammatory protein-2 (MIP-2), TNF-α, IL-6, IL-1β and IL-10 [20]. As acute STI is characterized with excessive inflammatory responses, we conjecture the herbs made up of Huanglian-Jie-Du-Tang which have a good effect on mitigating acute STI. Here, we explored the effect of heat-clearing, detoxifying and blood stasis removing decoction (HDBD), to which was added *Astragalus membranaceus, Garden burnet, Rheum officinale, Carthamus tinctorius L.* and *Borneol* to Huanglian-Jie-Du-Tang recipe on acute STI.

## Materials and methods

### HDBD preparation

HDBD contained 30 g *Coptis chinensis*, 10 g *Cortex Phellodendri*, 10 g *Scutellaria baicalensis*, 10 g *Fructus gardenia*, 20 g *Astragalus membranaceus*, 10 g *Garden burnet*, 10 g *Rheum officinale*, 5 g *Carthamus tinctorius L.* and 3 g *Borneol* which were added to 500 ml water and boiled until the volume was 100 ml.

### Animals

Thirty male Sprague–Dawley (SD) rats (250–300 g) aged 6–7 weeks were provided by Nanjing Junke Biological Co., LTD. (Jiangsu Province, China) for use in the present study. All animals were maintained in a temperature-controlled condition (24 ± 2°C), with free access to water and chow in a 12-h/12-h light/dark cycle.

### Cell lines and culture

The human dermal microvascular endothelium cell line HMEC-1 was purchased from American Type Culture Collection (ATCC, Manassas, VA, U.S.A.). The rat vascular endothelium cell line RAOEC was purchased from Shanghai Jingkang Biological Co., LTD (Shanghai, China). HMEC-1 and RAOEC cells were cultured in MCDB131 (without l-Glutamine), with 10 ng/ml Epidermal Growth Factor (EGF), 1 µg/ml Hydrocortisone, 10 mM Glutamine and 10% (v/v) fetal bovine serum (FBS), all obtained from ATCC.

For HDBD intervention, the cells were incubated with 3:1 (v/v) culture medium/HDMD for 24 h.

### Cell transfection

The miR-26b-5p mimic/inhibitor (mimic/inhibitor-miR-26b-5p), COX2 overexpressing plasmid (OE-COX2) and the negative control vectors (mimic/inhibitor-NC and OE-NC) were designed and synthesized by GenePharma Co., LTD. (Shanghai, China). Lipofectamine™ 2000 transfection reagent (Invitrogen, Waltham, MA, U.S.A.) was applied for cell transfection based on the instruction book.

### *In vivo* experimental design and treatment

Following 1 week of acclimatization, the SD rats were divided randomly into three groups: sham group (saline 0.5 ml), model group (saline 0.5 ml) and HDBD group (HDBD 0.5 ml), with 10 rats in each group. One percent pentobarbital was applied to anesthetize rats intraperitoneally. The hair removal of medial thigh of the knee joint was carried out by using the depilatory cream. Then, the STI models were established with the help of a self-made hammer according to a previous study [3]. A hammer weighing ∼270 g with a bottom-surface radius of 0.6 cm, was dropped from a height of 30 cm to beat the medial thigh (1 cm from the knee joint) on the right side. Rats in the sham group were hit by a marker pen from same height. No bone fracture was caused. Following 30 min of the strike, rats in the sham and model groups were embrocated 0.5 ml saline on the injury area, while HDBD group was embrocated 0.5 ml HDBD once a day for a total of 5 days. At 1, 3, 6 and 10 days after the strike, the temperatures of the healthy side and the affected side were measured using an infrared thermometer. Then, the rats were killed by exsanguination, and the injured muscle tissues were collected and washed with ice-cold saline, and submitted for testing.

### Histopathology studies

The muscle tissues were fixed in 4% paraformaldehyde, and embedded in paraffin. The 5-μm sections were cut and stained with Hematoxylin–Eosin (HE). Slides were scanned and images were taken under an optical microscope (200×; Olympus Corporation, Tokyo, Japan).

### Enzyme-linked immunosorbent assay

Following being homogenized with PBS, the levels of TNF-α, IL-6, IL-1 and PGE2 in the muscle tissues were measured by using enzyme-linked immunosorbent assay (ELISA) Kits (Abcam, Cambridge, MA, U.S.A.), as well as in cell supernatants in the light of the manufacturer’s protocols.

### Real-time quantitative polymerase chain reaction

Total RNA was extracted from muscle tissues and cells with TRIzol reagent (Life Technologies, Waltham, MA, U.S.A.) referring to the manufacturer’s manual. Then, the cDNA was produced using a PrimeScript™ First Strand cDNA Synthesis Kit (Takara, Dalian, China). After that, the cDNA was served as substrate for quantitative polymerase chain reaction (qPCR) with SYBR Premix ExTaq (Takara) on an ABI 7900 system (Applied Biosystems, Foster City, CA, U.S.A.). 2^−ΔΔ*C*_t_^ method was used to calculate the relative levels of mRNAs after being normalized to that of the expression level of glyceraldehyde 3-phosphate dehydrogenase (GAPDH).

Homo-COX2-sense-5′-AGTCCCTGAGCATCTACGGT-3′,
COX2-antisense-5′- AAAGGTGTCAGGCAGAAGGG-3′;
Rattus-COX2-sense-5′- AAAGGTGTCAGGCAGAAGGG-3′
COX2-antisense-5′- GGGTGGGCTTCAGCAGTAAT-3′;
Homo-GAPDH-sense-5′- CACTAGGCGCTCACTGTTC-3′,
GAPDH-antisense-5′- GAGGGATCTCGCTCCTGGAA-3′;
Rattus-GAPDH-sense-5′- AGTGCCAGCCTCGTCTCATA-3′,
GAPDH-antisense-5′- GATGGTGATGGGTTTCCCGT-3′.

### Western blotting

Protein samples isolated from tissues and cells were separated by the sodium dodecyl sulfate/polyacrylamide gel electrophoresis (SDS/PAGE) and were transferred on to the PVDF membranes (Millipore, Billerica, MA, U.S.A.). After that, the membranes were incubated with the indicated primary antibodies, including anti-COX2 (cat. no. ab15191) and anti-GAPDH (cat. no. ab181602, 1:5000) antibodies, all purchased from Abcam at 4°C overnight. Then, the membranes were incubated with the horseradish peroxidase (HRP)-conjugated indicated secondary antibodies (1:5000) for 1 h at room temperature. Then, the protein expression signals were measured with the help of an ECL kit (Millipore). ImageJ software (version 1.48; National Institutes of Health) was applied for protein quantification.

### Luciferase gene reporter assay

Luciferase reporter plasmids including the 3′-UTR of COX2 (called as COX2-WT) and the empty luciferase vector were purchased from the Shanghai GenePharma Co., Ltd (Shanghai, China). Mutations were made within the predicted target sites between miR-26b-5p and COX2, which was named as COX2-MUT. To start the luciferase gene reporter assay, cells were seeded in 96-well plates and co-transfected with the COX2-WT/COX2-MUT and mimic-NC/mimic-miR-26b-5p with the help of FuGENE FuGENE® HD transfection reagent (Promega, Madison, WI, U.S.A.). After 48 h of the above treatment, the luciferase activity in each well was measured by using Luciferase Assay Kit (Promega).

### Statistical analysis

All data are expressed as mean ± SD. Statistical analyses were performed using one-way analysis of variance and Student’s *t* tests with the help of GraphPad Prism 5.0 software. Value of *P*<0.05 was considered significant difference.

## Results

### HDBD intervention assuaged acute STI with reduced inflammation

**Figure 1:**
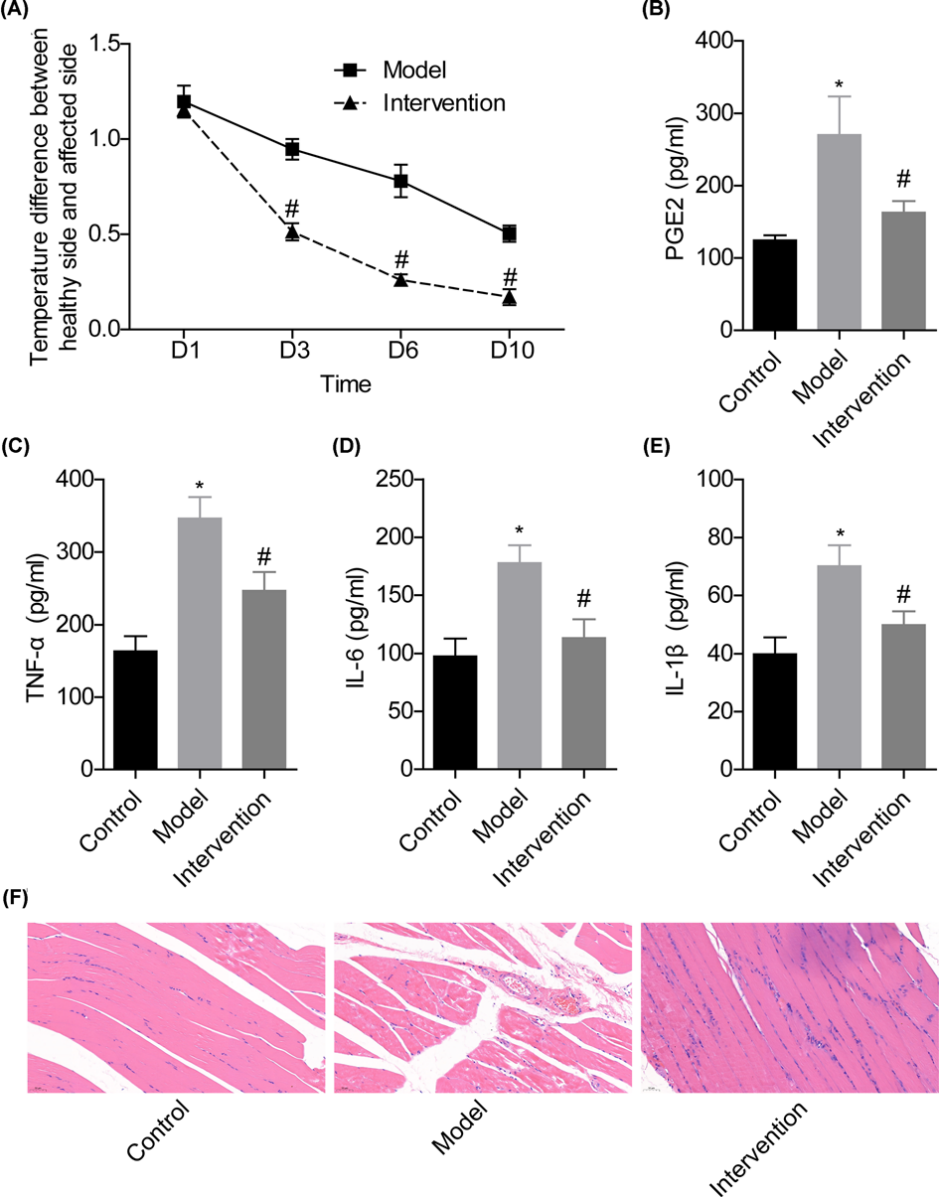
HDBD intervention assuaged acute STI and reduced inflammation in rat models (**A**) The temperature difference between the healthy side and affected side of rats from the model group and intervention group. (**B**–**E**) The levels of PGE2, TNF-α, IL-6 and IL-1β in the affected muscle tissues were measured by ELISA. (**F**) HE staining (200×) to assess the (*P<0.05, vs. control group; ^#^*P*<0.05, vs. model group).

Compared with the model group, the temperature difference between the healthy side and affected side was reduced when the STI mice were given HDBD treatment (Figure 1A). In addition, HDBD treatment significantly reduced the levels of inflammatory factors, including TNF-α, IL-6, PGE2 and IL-1β in the muscle tissues caused by hammer beating (Figure 1B–E). Moreover, tissue edema and the inflammatory cell infiltration were observed in the model group, which were mitigated following HDBD intervention, as shown in Figure 1F. These results demonstrated that HDBD could assuage acute STI and inhibit the excessive inflammatory responses.

### HDBD intervention increased miR-26b-5p expression and reduced COX2 expression in acute STI rats

**Figure 2:**
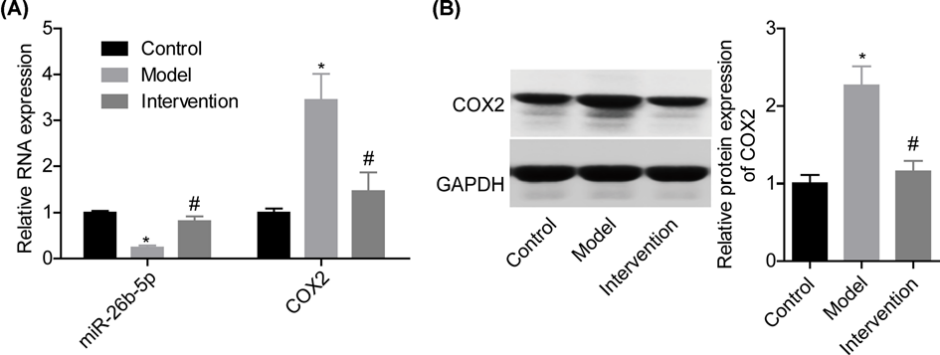
HDBD intervention increased miR-26b-5p expression and reduced COX2 expression in acute STI rats The affected muscle tissues were obtained from rats in control, model and intervention groups, the (**A**) qPCR and (**B**) Western blotting assays were carried out to detect the expression of miR-26b-5p and/or COX2 (*P<0.05, vs. control group; ^#^*P*<0.05, vs. model group).

To uncover whether miR-26b-5p/COX2 axis is involved in HDBD-mediated STI alleviation, we detected the effects of HDBD intervention on the expression of miR-26b-5p and COX2 in the muscle tissues from the affected thigh. Compared with the sham group, miR-26b-5p level was reduced in the model group while COX2 expression was increased, whereas HDBD intervention reversed these tendencies (Figure 2A,B). This result suggested that miR-26b-5p and COX2 might be involved in HDBD role in assuaging acute STI.

### COX2 was a target of miR-26b-5p in both human and rat species

**Figure 3:**
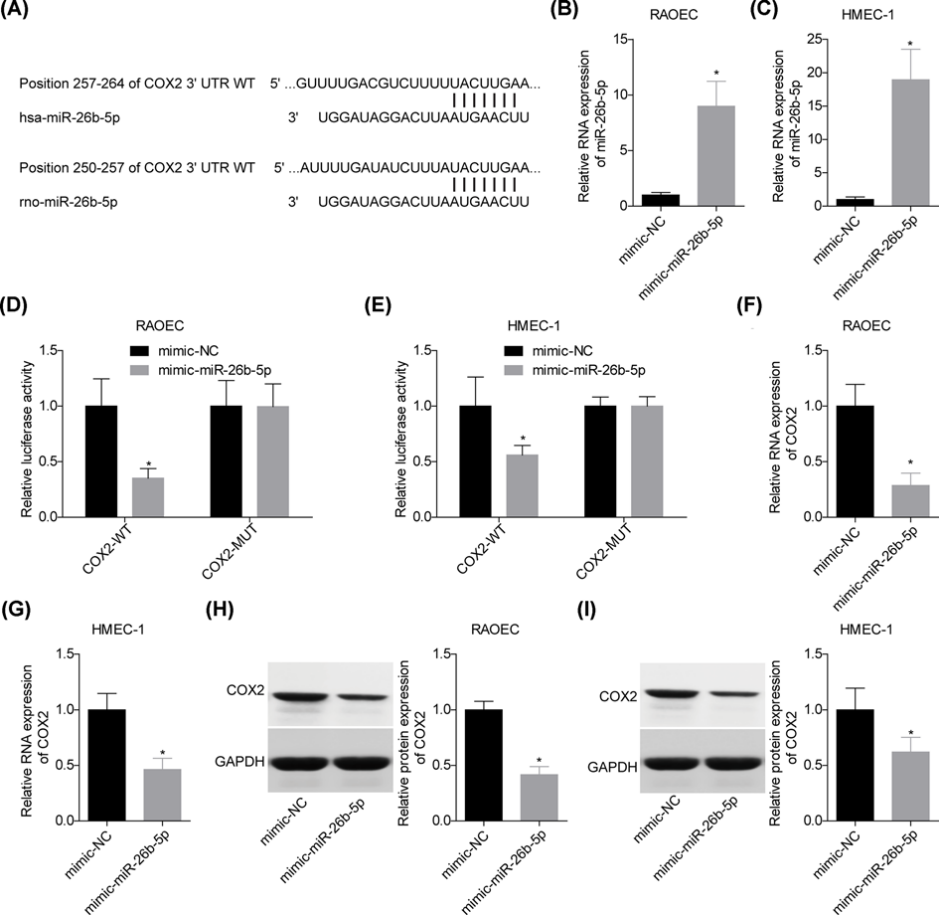
miR-26b-5p targeted COX2 in both human and rat species (**A**) The putative binding sites between miR-26b-5p and the 3′UTR of COX2. (**B,C**) qPCR was performed to test the expression of miR-26b-5p following 48 h of cell transfection with mimic-NC or mimic-miR-26b-5p. (**D,E**) The luciferase activity of COX2-WT was tested following 48 h of cell transfection with mimic-NC or mimic-miR-26b-5p. (**F**–**I**) qPCR and Western blotting assays were used to detect the mRNA and protein levels of COX2 following 48 h of cell transfection with mimic-NC or mimic-miR-26b-5p (*n*=3, *P<0.05, vs. mimic-NC group).

Next, we explored the relationship between miR-26b-5p and COX2 *in vitro*. Figure 3A showed the putative binding sites between miR-26b-5p and the 3′UTR of COX2 in human and rats. Compared with the mimic-NC group, transfection with mimic-miR-26b-5p induced an obvious increase in miR-26b-5p level in RAOEC and HMEC-1 cells (Figure 3B,C). The luciferase gene reporter assay showed that up-regulation of miR-26b-5p caused a dramatic reduction in the luciferase activity, whereas it was abolished when the binding sites of miR-26b-5p in the 3′UTR of COX2 were mutated (Figure 3D,E). In addition, miR-26b-5p overexpression apparently decreased COX2 expression at both mRNA and protein levels in RAOEC and HMEC-1 cells (Figure 3F–I). These findings confirmed that COX2 was a target of miR-26b-5p in human and rat species.

### miR-26b-5p repressed inflammatory responses via targeting COX2 *in vitro*

**Figure 4:**
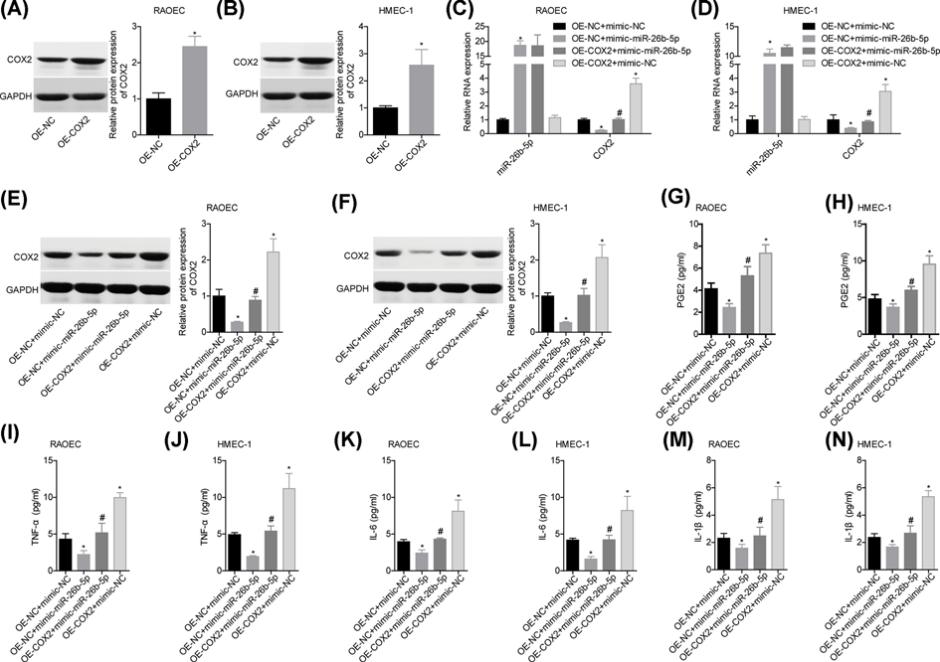
miR-26b-5p repressed inflammatory responses via targeting COX2 *in vitro* (**A,B**) Following 48 h of cell transfection with OE-NC/OE-COX2, RAOEC and HMEC-1 cells were collected for Western blotting assay to detect the protein level of COX2. Then, cells in OE-NC+mimic-NC, OE-NC+mimic-miR-26b-5p, OE-COX2+mimic-miR-26b-5p and OE-COX2+mimic-NC groups were submitted to the following assays. (**C,D**) The levels of miR-26b-5p and COX2 mRNA in cells were tested by using qPCR. (**E,F**) The protein level of COX2 was determined by Western blotting. (**G**–**N**) The levels of PGE2, TNF-α, IL-6 and IL-1β in the supernatants of cells were measured by ELISA (*n*=3, *P<0.05, vs. OE-NC+mimic-NC group; ^#^*P*<0.05, vs. OE-NC+mimic-miR-26b-5p group).

We then investigated the role of miR-26b-5p/COX2 axis in the inflammatory reactions *in vitro*. COX2 expression was significantly increased following cell transfection with OE-COX2 in RAOEC and HMEC-1 cells (Figure 4A,B), whereas this effect was abolished after miR-26b-5p overexpression (Figure 4C–F). In addition, miR-26b-5p overexpression reduced the levels of PGE2, TNF-α, IL-6 and IL-1β in the supernatants of RAOEC and HMEC-1 cells, whereas COX2 up-regulation weakened this role (Figure 4G–N). These results demonstrated that miR-26b-5p repressed inflammatory responses via targeting COX2.

### HDBD intervention inhibited inflammation via modulating miR-26b-5p/COX2 axis *in vitro*

**Figure 5:**
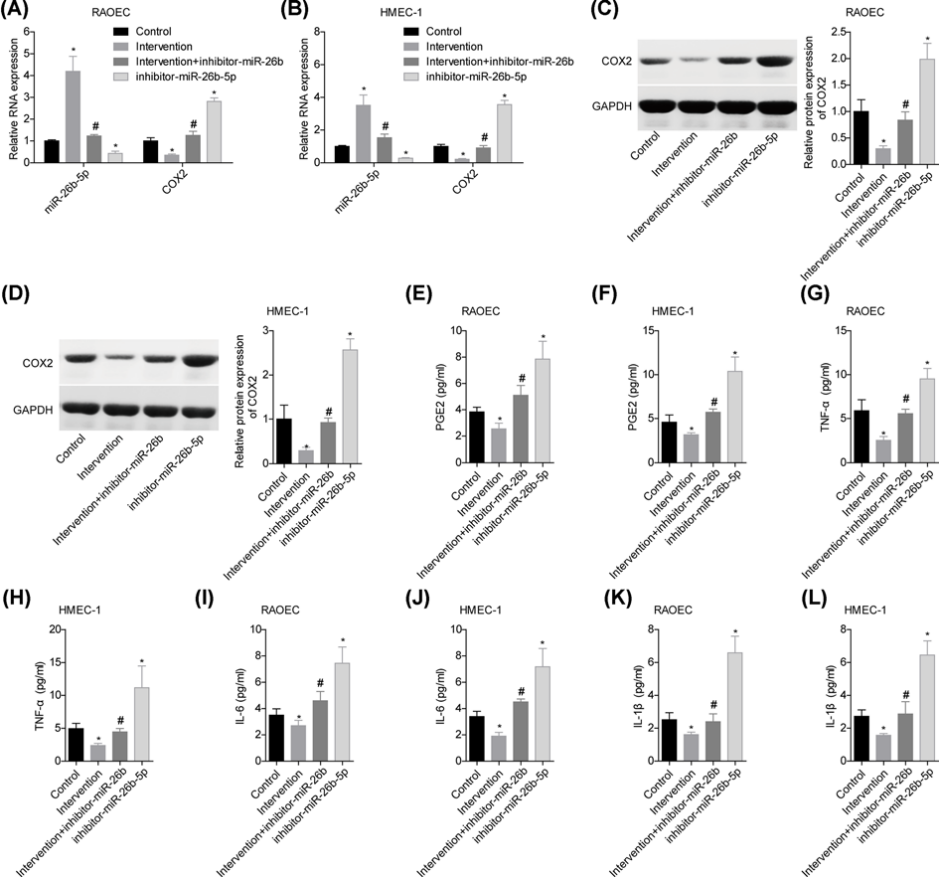
HDBD intervention inhibited inflammation via modulating miR-26b-5p/COX2 axis *in vitro* RAOEC and HMEC-1 cells in the control, intervention, intervention+inhibitor-miR-26b-5p and inhibitor-miR-26b-5p groups were submitted to the following experiments. (**A,B**) The levels of miR-26b-5p and COX2 mRNA in cells were tested by using qPCR. (**C,D**) The protein level of COX2 was determined by Western blotting. (**E**–**L**) The levels of PGE2, TNF-α, IL-6 and IL-1β in the supernatants of cells were measured by ELISA (*n*=3, *P<0.05, vs. control group; ^#^*P*<0.05, vs. intervention group).

Next, we explored whether miR-26b-5p/COX2 axis was involved in HDBD-mediated STI improvement *in vitro*. HDBD intervention increased miR-26b-5p expression and decreased COX2 expression in RAOEC and HMEC-1 cells, whereas inhibitor-miR-26b-5p reversed this effect (Figure 5A–D). Accordingly, the reduced levels of PGE2, TNF-α, IL-6 and IL-1β induced by HDBD treatment were reversed when miR-26b-5p was down-regulated (Figure 5E–L). These results suggested that HDBD intervention inhibited inflammation via modulating miR-26b-5p/COX2 axis.

## Discussion

The early phase of STI is featured with excessive production of pro-inflammatory factors and successive secondary damage [21]. Although inflammation is necessary to the healing process and benefits wound healing [22], it is indeed harmful when inflammatory responses get out of control during acute STI [23]. In the current study, we explored the function and mechanism underlying HDBD in the treatment of acute STI. The *in vivo* assay showed that HDBD intervention significantly improved the acute STI through inhibiting the excessive inflammatory responses. The *in vitro* assay demonstrated that HDBD served as a regulator of miR-26b-5p/COX2 axis to inhibit inflammatory responses.

Evidence has demonstrated that several kinds of TCM exert good effects on alleviation of acute STI. Hydroxysafflor yellow A, the effective flavonoid of dried safflower, attenuated pathologic changes in strike-induced soft tissue inflammation [3]. Wang et al. [23] found that Xiangqing Anodyne Spray suppressed STI-mediated muscle swelling, pro-inflammatory mediator productions, oxidative stress and severe pathological changes in the injured muscle tissue. Huanglian-Jie-Du-Tang, a heat-clearing and detoxifying decoction exerts an inhibitory role in inflammatory responses in different kinds of diseases, such as arthritis [24], liver injuries [25] and polymicrobial sepsis [26]. On the basis of Huanglian-Jie-Du-Tang, we added *Astragalus membranaceus, Garden burnet, Rheum officinale, Carthamus tinctorius L.* and *Borneol* to form HDBD. Due to the effects of removing blood stasis, stopping blood and/or protecting brain from injury of *Fructus gardenia, Garden burnet, Rheum officinale, Carthamus tinctorius L.* and *Borneol* [27–30], we speculated that HDBD might play a role in anti-inflammation and removing blood stasis. As expected, our results showed that HDBD intervention significantly assuaged the tissue edema and inflammatory cell infiltration in the acute STI model of rats induced by strike, accompanied by the reduced levels of pro-inflammatory factors, such as TNF-α, IL-6, IL-1β and PGE2.

COX is an enzyme which converts arachidonic acid into PGs, has been found to have two isoforms, COX1 and COX2. COX2 is responsible for production of large amounts of pro-inflammatory PGs at the inflammatory site, especially PGE-2, which can work synergistically with other inflammatory factors to exacerbate inflammatory responses [31,32]. In the present study, our results demonstrated that COX2 level was significantly increased in rat injured tissues induced by strike, and was rescued following HDBD administration, indicating that COX2 might be involved in HDBD-mediated mitigation of acute STI. Further exploration showed that COX2 was a target of miR-26b-5p in both human and rat cells. MiR-26b-5p level was demonstrated to be increased in subcutaneous adipose tissue underneath lesional psoriasis skin [33]. Moreover, it has been reported that miR-26b-5p binds to the neutral cholesterol ester hydrolase 1, an enzyme essential for cholesterol efflux which is of importance in the pathogenesis of atherosclerosis as its defection induces excess cholesterol accumulation and foam cells formation, linking miR-26b-5p to inflammation [33–35]. In the current study, we observed that miR-26b-5p level was significantly decreased in rat injured tissues, and was rescued following HDBD administration, which showed opposite to the expression level of COX2. Moreover, we found that miR-26b-5p decreased COX2 expression to repress the production of PGE2, TNF-α, IL-1β and IL-6. However, COX2 overexpression impaired the anti-inflammatory effects of miR-26b-5p *in vitro*, implying that miR-26b-5p inhibited inflammation by targeting COX2.

Additionally, we investigated whether miR-26b-5p/COX2 axis was involved in HDBD intervention-mediated inflammation repression. The results showed that inhibition of miR-26b-5p dramatically reversed the reductions in the production of PGE2, TNF-α, IL-1β and IL-6 in RAOEC and HMEC-1 cells induced by HDBD treatment. These results illustrated that HDBD intervention inhibited inflammation via regulating miR-26b-5p/COX2 axis.

In conclusion, the present study uncovered that HDBD could relieve acute STI via modulating miR-26b-5p/COX2 axis to inhibit inflammatory responses. The current study shows that HDBD may be a new effective drug for the treatment of acute STI through inhibiting the excessive inflammation. Collectively, our findings demonstrate the theoretical basis for the clinical use of HDBD in treating acute STI, and improve our understanding of the occurrence and development in acute SCI.

## Data Availability

All supporting data are included within the main article and its supplementary files.

## Abbreviations

ATCC: American Type Culture Collection
COX2: cyclo-oxygenase 2
GAPDH: glyceraldehyde 3-phosphate dehydrogenase
HDBD: heat-clearing, detoxifying and blood stasis removing decoction
IL: interleukin
PGE2: prostaglandin E2
SD: Sprague–Dawley
STI: soft tissue injury
TCM: traditional Chinese medicine
